# Fusion-Related Host Proteins Are Actively Regulated by NA during Influenza Infection as Revealed by Quantitative Proteomics Analysis

**DOI:** 10.1371/journal.pone.0105947

**Published:** 2014-08-25

**Authors:** Zhiwei Sui, Bo Wen, Zhimin Gao, Quanjiao Chen

**Affiliations:** 1 State Key Laboratory of Virology, Center for Emerging Infectious Diseases, Wuhan Institute of Virology, Chinese Academy of Sciences, Wuhan, Hubei, China; 2 National Institute of Metrology, Beijing, China; 3 BGI-Shenzhen, Shenzhen, China; The University of Chicago, United States of America

## Abstract

Three recombinant influenza A viruses with different neuraminidases (NAs) in the background of A/PR/8/34 (PR8), named rPR8-H5N1NA, rPR8-H9N2NA, and rPR8-H1N1NA, derived from H5N1, H9N2, H1N1 (swine) viruses, respectively, were constructed. We performed a quantitative proteomics analysis to investigate differential protein expression in Madin-Darby canine kidney (MDCK) cells infected with recombinant and wild-type influenza viruses to determine whether NA replacement would alter host cell gene expression. Using matrix-assisted laser desorption/ionization time-of-flight mass spectrometry (MALDI-TOF-TOF MS) and two-dimensional gel electrophoresis (2-DE), we identified 12 up-regulated and 49 down-regulated protein spots, including cytoskeletal proteins, molecular biosynthesis proteins, ubiquitin-proteasome pathway proteins, and heat shock proteins. The most significant changes in infected cells were observed for molecular biosynthesis proteins. We found more differentially expressed protein spots in cells infected with rPR8-H5N1NA or rPR8-H9N2NA viruses than cells infected with wild-type virus. Many of those proteins are postulated to be involved in cell-cell fusion, but the full mechanism remains to be explored. Meanwhile, our data demonstrate that the wild-type virus has evolutionary advantages over recombinant viruses.

## Introduction

Virus evolution is inseparable from virus–host interactions, and there have been many studies focused on the interactions between influenza viruses and their hosts in the past several years [1]. Proteomic studies have made it possible to elucidate the complex relationships between viruses and their hosts, and many proteome analyses have been performed to determine how protein expression changes following influenza viral infection [2]-[4]. Liu et al. focused on human cell lines infected with the avian H9N2 influenza virus and investigated a possible adaptation mechanism of avian influenza virus [4]. Baas et al. [3] employed a macaque animal model infected with the influenza A virus and combined functional non-gel based proteome approaches with mRNA microarrays. Mayer et al. [2] identified cellular factors associated with native viral ribonucleoproteins (vRNPs) and viral polymerase complexes. However, all of these studies focused on comparisons between human cells infected with wild-type influenza virus and mock-infected cells. It is still unclear whether recombinant viruses with neuraminidases (NA) gene replacements will differentially alter protein expression.

We obtained three recombinant viruses with NAs from a highly pathogenic avian H5N1 virus, the pathogenic H9N2 virus, and the 2009 human pandemic H1N1 (swine) virus in the background of the A/PR/8/34 (PR8) (H1N1) virus. Previous studies of these recombinant viruses indicated that they had different influenza virus infection initiation and virus release rates in vitro. Each recombinant virus and the wild-type virus induced cell-cell fusion in Madin-Darby canine kidney (MDCK) cells at 12 h post-infection (p.i.) [5], while recombinant viruses rPR8-H5N1NA and rPR8-H9N2NA induced different degrees of cell-cell fusion compared with wild-type virus in MDCK cells at 6 h p.i., suggesting that differential protein expression between rPR8-H5N1NA or rPR8-H9N2NA and wild-type virus may be associated with cell fusion. To further explore cell responses to NA replacement and cell fusion-related protein expression, we harvested cells at 6 h p.i., performed two-dimensional gel electrophoresis (2-DE), and analyzed distinct spots with mass spectrometry.

In nature, only a few virus strains have survived in hosts or caused pandemics. A virus strain is often replaced by others after it is prevalent for a period of time. Actually, influenza viruses and hosts can co-adapt and co-evolve. When a virus replicates stably in the host without influencing the host life cycle, it would favor stable virus survival in hosts for an extended period of time. It is unknown whether proteins will be differentially expressed in MDCK cells infected with recombinant viruses versus wild-type virus and which proteins will be affected. These data will be helpful in understanding the function of NA in influenza viruses and the pattern of viral evolution.

## Materials and Methods

### Virus, cell culture, virus infection, and sample preparation

The recombinant viruses rPR8-H5N1NA, rPR8-H9N2NA, and rPR8-H1N1NA and the NA gene from influenza viruses A/H5N1, A/H9N2, and swine A/H1N1 virusA/PR/8/34 (PR8-wt) were conserved by the Wuhan Institute of Virology, Chinese Academy of Sciences and stored at −80°C.

MDCK cells were incubated in a 35 mm × 10 mm cell culture dish in minimal essential medium (MEM) containing 10% fetal calf serum (FCS) at 37°C in 5% CO_2_. MDCK cell monolayers were inoculated with diluted virus at a multiplicity of infection (MOI) of 0.1, and the inoculum was removed after incubation at 37°C for 1 h. The cells were washed and overlaid with 3 ml MEM containing 1.0 µg/ml trypsin. The total cellular proteins were extracted at 6 h p.i.

### Protein extraction

Proteins were prepared as follows: cells were lysed in lysis buffer containing 7 M urea, 2% CHAPS (3-[(3-cholamidopropyl)-dimethylammonio]-1-propanesulfonate), 2 M thiourea, 20 mM Tris–HCl (pH8.5), and phenylmethylsulfonyl fluoride solution (Amresco, Solon, OH, USA) and sonicated on ice (40 W, 12 s duration, 10 times, 2 min intervals) and centrifuged (12,000×g for 20 min at 4°C). The supernatant was transferred to a new centrifuge tube and acetone was added. The mixture was then precipitated overnight at −20°C and centrifuged the next day. The precipitate was harvested and stored at −80°C until use. The samples were prepared in triplicate.

### 2-DE

The precipitate sample was dissolved in rehydration buffer containing 7 M urea, 2% CHAPS, and 2 M thiourea, then centrifuged at 12,000×g for 20 min at 4°C. The protein concentration was determined using a 2 D Quant kit according to the manufacturer's instructions (Amresco). Proteins were first separated on 18-cm pH 4-7 immobilized pH gradient (IPG) gel strips. After isoelectric focusing, the IPG strip was equilibrated in equilibration buffer containing 2% sodium dodecyl sulfate (SDS), 50 mM Tris–HCl (pH 8.8), 6 M urea, 30% (vol/vol) glycerol, 0.002% bromophenol blue, and 100 mM dithiothreitol (Amresco).

After equilibration, the strips were loaded onto 12.5% (w/v) polyacrylamide gels. Proteins were separated by running the gels at 2 W/gel for 45 min and then at 18 W/gel at 10°C until the end. Finally, the gels were stained with Coomassie Brilliant Blue G-250 overnight and rinsed with deionized water.

### Image analysis

Images of gels were obtained at 150 dpi (dots/in) using a scanner (Powerlook1100, UMAX, Dallas, TX, USA) and analyzed using ImageMaster 2D Platinum 5.0 software (GE Healthcare, Waukesha, WI, USA). Spots were detected on the gel by eye and a 2DElite automatic spot detection program (GE Healthcare) that calculated spot volumes relative to the background and normalization. The volume percentages of each spot were determined by comparison of the spot volume to the total volume in the 2-DE gel.

### Protein identification and function analysis

After gel image analysis, the differentially expressed protein spots were excised and enzymolysized using trypsin. The peptide masses were measured using a matrix-assisted laser desorption/ionization time-of-flight (MALDI-TOF-TOF) mass spectrometer (ABI4700 Applied Biosystems, Foster City, CA, USA). The threshold criteria and settings were: mass range of 700-3200 Da (optimal resolution: 1500 Da), a laser frequency of 50 Hz, a repetition rate of 200 Hz, an ultraviolet (UV) wavelength of 355 nm, and acceleration voltage of 20,000 V.

Combined MS and MS/MS spectra were submitted to MASCOT (http://www.matrixscience.com) and searched against the National Center for Biotechnology Information non-redundant (NCBInr) database (release date, January 7, 2012) using the following parameters: taxonomy of Metazoa (Animals), trypsin digest with one missing cleavage, fixed modifications of carbamidomethyl (C), variable modification of Oxidation(M), peptide mass tolerance of ±50 ppm, fragment mass tolerance of ±0.5 Da. MASCOT protein scores (based on combined MS and MS/MS spectra) of greater than 77 were considered statistically significant (*p*≤0.05). Individual MS/MS spectra with a statistically significant (confidence interval≥95%) ion score (based on MS/MS spectra) were accepted.

Blast2GO software was used to assign gene ontology (GO) terms for the identified proteins sequences against the NCBInr database (E value < 1e−5) [6].

### Quantitative real-time reverse transcription polymerase chain reaction (qRT-PCR)

According to the corresponding gene sequences of MS-identified proteins, specific primers were designed using Beacon Designer 7.9 analysis software (Premier Biosoft International, Palo Alto, CA, USA). Total RNA was extracted using an RNeasy Mini Kit (QIAGEN, Venlo, The Netherlands) according to the manufacturer's protocol. Six important differentially expressed proteins were examined to detect the corresponding mRNA levels to validate protein expression changes. qRT-PCR was performed as described previously [7]. Total cDNA was produced by reverse transcription (RT) of equal amounts of RNA (1 µg) using random primers (Roche, Basel, Switzerland) according to the manufacturer's protocols. qRT-PCR was performed on a LightCycler 480 (Roche) instrument according to the instructions of the LightCycler FastStart DNA Master plus SYBR Green kit (BioRad, Hercules, CA, USA) in a total volume of 20 µL. Subsequently, melting curve analysis and quantitative analysis of the data were performed using the Roche LightCycler 480 software version 1.5.0. Each sample was run in triplicate. The β-actin mRNA level was used as the reference for normalization.

### Western blot analysis

MDCK cells were plated into six-well plates. Twenty-four hours after plating, the cells were infected with influenza virus. The cells were harvested at 6 h p.i. and washed twice with cold phosphate-buffered saline (PBS) and lysed with a cell lysis buffer (Cell Signaling Technology, Beverly, MA, USA). The cell lysates were analyzed using 12% SDS-polyacrylamide gel electrophoresis (PAGE), blotted onto polyvinylidene fluoride (PVDF) membranes, and then immunoblotted with primary antibodies (Abcam, Cambridge, UK). A horseradish peroxidase (HRP)-conjugated goat anti-rabbit immunoglobulin G (Santa Cruz Biotechnology, Santa Cruz, CA, USA) was used as the secondary antibody. Finally, the membranes were visualized using SuperSignal West Pico Chemiluminescent substrate (Pierce, Rockford, IL, USA). Each reaction was performed in triplicate.

### Indirect immunofluorescence assay (IIFA)

MDCK cell monolayers were seeded on glass coverslips were inoculated with a virus solution which was removed after 1 hour of incubation, and the cells were incubated at 37°C for additional 6 h. Then the cultures were fixed with 4% paraformaldehyde, permeabilized with 0.5% Triton X-100, blocked with 5% non-fat milk, and stained with goat polyclonal antibody to UBE2NL. Next, the fluorescein isothiocyanate (FITC)-conjugated anti-goat IgG (Millipore) secondary antibodies were added and then stained with Hoechst 33258 for 10 min. Fluorescent image analysis was performed on a Leica laser scanning confocal microscope with associated software as described previously [5].

### Statistical analysis

The relative spot intensities corresponding to the levels of abundance of proteins in the influenza virus-infected and mock-infected groups or recombinant viruses-infected and PR8-wt-infected groups were compared. The criterion for a significant change in protein expression in the infected cells compared to that in mock-infected or recombinant virus-infected cells to that in PR8-wt-infected cells was considered significant when it was >2.0 or <0.5 (both *p*≤0.05).

## Results and Discussion

### 2-DE profiles of influenza-infected MDCK cells

**Figure 1:**
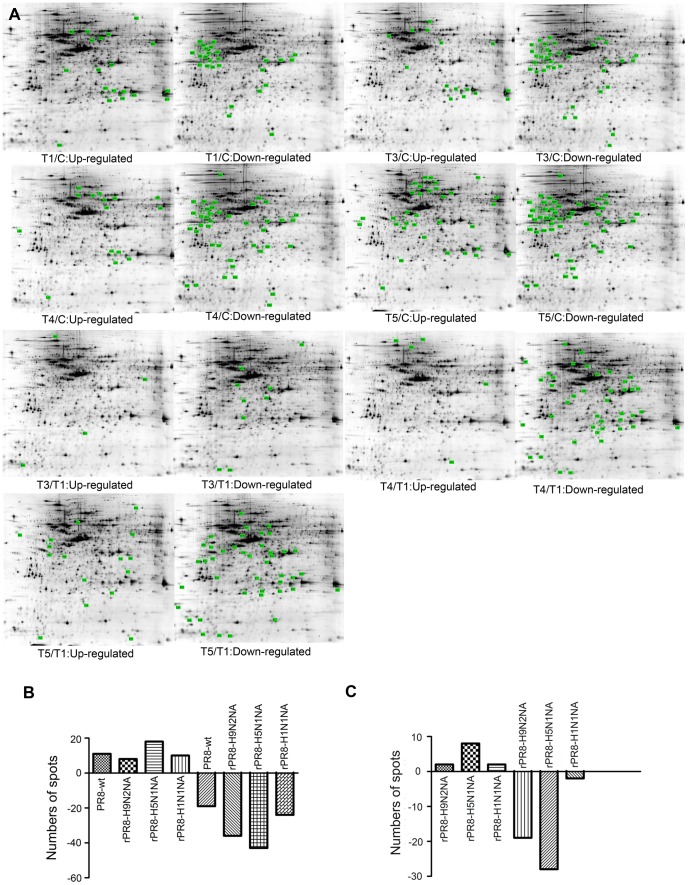
Protein expression profiles of the influenza- and mock-infected MDCK cells. Cell lysates (120 µg) were separated on 13-cm (isoelectric point [pI] 4–7) linear gradient IPG strips using 12.5% SDS-PAGE. Differentially expressed protein spots are indicated with green squares. (A) Representative 2-DE gels of influenza- and mock-infected MDCK cells. T1/C: PR8-wt infected/mock infected, T3/C: rH1N1NA infected/mock infected, T4/C: rH9N2NA infected/Mock infected, T5/C: rH5N1NA infected/mock infected, T3/T1: rH1N1NA infected/PR8-wt infected, T4/T1: rH9N2NA infected/PR8-wt infected, T5/T1: rH5N1NA infected/PR8-wt infected. (B) Numbers of differentially expressed protein spots detected by 2-DE in virus-infected MDCK cells compared with mock-infected MDCK cells. The number of spots ≥0 indicated the proteins were upregulated, and the number <0 indicated the proteins were downregulated. (C) Numbers of differentially expressed protein spots detected by 2-DE in recombinant viruses compared with wild-type virus (wt-PR8)-infected MDCK cells.

Proteins were extracted from virus- and mock-infected MDCK cells at 6 h p.i. for 2-DE analysis. Based on the methodology of defined differential spots in the Materials and Methods section, the expression levels of 169 proteins in virus-infected cells were significantly altered (*p*<0.05) compared with mock-infected cells; among them, 47 were up-regulated and 122 were down-regulated (Fig. 1A, B).

To determine the effect of NA replacement, we compared cells infected with recombinant and wild-type viruses. We found that 61 proteins in the infected cells differed between cells infected with wild-type virus versus those infected with recombinant viruses, including 12 significantly up-regulated proteins and 49 significantly down-regulated proteins (Fig. 1C). More differential spots were identified in cells infected with rPR8-H5N1NA or rPR8-H9N2NA recombinant viruses than those infected with other viruses (Fig. 1B). However, the number of differential spots in cells infected with rPR8-H1N1NA was almost equal to those in cells infected with PR8-wt (Fig. 1A, C).

### Mass spectrometry identification of differentially expressed proteins

**Figure 2:**
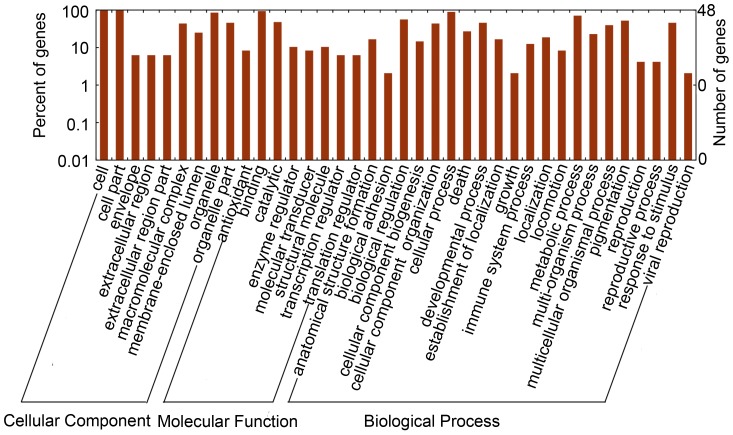
Classification of the identified proteins based on their functional annotations using Gene Ontology (GO) categories. The proteins were annotated into three main categories: cellular component, biological process, or molecular function. The Y-axis indicates the number and percentages of genes, the X-axis indicates the GO category.

To identify differentially expressed proteins in virus-infected MDCK cells, a total of 169 differential spots with >2-fold expression change were excised and subjected to MALDI-TOF-TOF mass spectrometry (MS). We successfully identified 64 differentially expressed protein spots. Thirty-six proteins were differentially expressed in the mock-infected cells and virus-infected cells (Table 1), including 19 significantly up-regulated proteins and 17 significantly down-regulated proteins. According to MASCOT and Blast 2GO analyses, the identified proteins included 5 cytoskeleton proteins, 16 macromolecular biosynthesis proteins, 5 stress response proteins, 2 signal transduction proteins, 2 metabolic process proteins, 5 viral proteins, and 1 other protein. According to the GO classification, the differentially expressed proteins were mainly concentrated in biological processes (Fig. 2).

**Table 1:** List of differentially expressed protein spots in influenza virus-infected and mock-infected MDCKs identified by MALDI-TOF/TOF.

Spot no.	Accession no.	Taxonomy	Description	Variation^a^	Best ion score	Coverage (%)	Theoretical Mr/pI	Localization	Peptides identified
**Cytoskeleton proteins**									
**I26**	gi|296202808	Canis lupus familiaris	uncharacterized protein	↓	39	30%	33853/8.31	cytoplasm	SQYEQLAEQNRK
**J11**	gi|359279916	Canis lupus familiaris	keratin 19	↑	53	56%	43858/5.16	cytoplasm	DAEAWFTSR
**M10**	gi|57106334	Canis lupus familiaris	keratin, type I cytoskeletal 18 isoform 1	↓	29	35%	48209/5.33	cytoplasm	VKLEAEIATYR
**N12**	gi|359323093	Canis lupus familiaris	keratin, type II cytoskeletal 8	↑	73	58%	54684/5.66	cytoplasm	SNIDNMFESYINNLR
**N26**	gi|114667511	Canis lupus familiaris	keratin, type I cytoskeletal 10 isoform 3	↑	0	20%	58416/5.09	cytoplasm	GSLGGGFSSGGFSGG
**Macromolecular biosynthesis**									
**G11**	gi|119586801	Homo sapiens	heterogeneous nuclear ribonucleoprotein C (C1/C2), isoform CRA_c	↓	41	30%	19079/10.22	nucleus	GFAFVQYVNER
**I17**	gi|57088769	Canis lupus familiaris	N(G),N(G)-dimethylarginine dimethylaminohydrolase 1	↓	28	27%	31629/5.94	cytoplasm	AQGDEVDFAR
**I19**	gi|50978728	Canis lupus familiaris	serine/threonine-protein phosphatase PP1-alpha catalytic subunit	↓	32	50%	38131/5.94	cytoplasm	ICGDIHGQYYDLLR
**I20**	gi|168277712	Canis lupus familiaris	uncharacterized protein	↓	61	39%	48760/5.38	nucleus	NLPLPPPPPPR
**I23**	gi|355712722	Canis lupus familiaris	uncharacterized protein	↓	55	42%	31507/4.79	nucleus	FIIENTDLAVANSIR
**I28**	gi|209736954	Canis lupus familiaris	Nascent polypeptide-associated complex subunit alpha	↓	102	23%	23376/4.57	nucleus	DIELVMSQANVSR
**I30**	gi|57086915	Canis lupus familiaris	protein DJ-1 isoform 1	↓	60	70%	20170/5.97	cytoplasm	GAEEMETVIPVDVMR
**J2**	gi|168277712	Canis lupus familiaris	uncharacterized protein	↑	61	30%	48760/5.38	nucleus	NLPLPPPPPPR
**J13**	gi|281338419	Canis lupus familiaris	Splicing factor	↑	34	61%	13350/10.12	nucleus	NPPGFAFVEFEDPR
**J15**	gi|119574194	Homo sapiens	heterogeneous nuclear ribonucleoprotein H1 (H), isoform CRA_b	↑	98	27%	44025/6.47	nucleus	HTGPNSPDTANDGFVR
**K20**	gi|210032390	Homo sapiens	protein SEC13 homolog isoform 2	↓	51	31%	34504/5.40	cytoplasm	NGGQILIADLR
**M2**	gi|359323746	Canis lupus familiaris	T-complex protein 1 subunit epsilon isoform 1	↓	43	28%	60015/5.58	cytoplasm	WVGGPEIELIAIATGGR
**M18**	gi|296207372	Canis lupus familiaris	uncharacterized protein	↓	45	22%	46415/4.90	nucleus	HPSKPDPSGECNPDLR
**N18**	gi|355722466	Mustela putorius furo	serine/threonine kinase receptor associated protein	↑	87	47%	39094/5.54	nucleus	FSPDGELYASGSEDGTLR
**N20**	gi|57088769	Canis lupus familiaris	N(G),N(G)-dimethylarginine dimethylaminohydrolase 1	↑	28	27%	31629/5.94	cytoplasm	AQGDEVDFAR
**N33**	gi|21465649	Canis lupus familiaris	Proteasome subunit beta type	↑	38	51%	22118/4.90	cytoplasm	LAAIAESGVER
**Stress response**									
**K1**	gi|296474454	Canis lupus familiaris	uncharacterized protein	↓	74	35%	78895/5.18	mitochondrion	HFSVEGQLEFR
**K30**	gi|345803242	Canis lupus familiaris	peroxiredoxin-6	↓	107	60%	25052/5.71	lysosom	PGGLLLGDEAPNFEANTTIGR
**K33**	gi|359322074	Canis lupus familiaris	peroxiredoxin-2-like isoform 1	↓	112	41%	22112/5.23	cytoplasm	EGGLGPLNIPLVADVTR
**M25**	gi|33987931	Homo sapiens	HSP90AB1 protein	↓	50	27%	40270/4.89	mitochondrion	HFSVEGQLEFR
**N3**	gi|345797614	Canis lupus familiaris	60 kDa heat shock protein, mitochondrial	↑	129	42%	61128/5.78	mitochondrion	AAVEEGIVLGGGCALLR
**Signal transduction**									
**I24**	gi|73946797	Canis lupus familiaris	annexin A1	↓	29	16%	38887/5.84	cytoplasm	TPAQFDADELR
**I29**	gi|55742853	Canis lupus familiaris	annexin A4	↓	107	32%	36075/5.72	cytoplasm	GGTVKPASGFSATEDAQTLR
**Metabolism associated proteins**									
**J14**	gi|169403978	Canis lupus familiaris	glutathione peroxidase 1	↑	37	43%	22607/6.15	cytoplasm	FLVGPDGVPVR
**N32**	gi|345789767	Canis lupus familiaris	inosine triphosphate pyrophosphatase	↑	21	51%	23485/6.17	cytoplasm	SAYALCTFAFSTGDPSEPVR
**Viral proteins**									
**J8**	gi|312436295	Influenza A virus	nonstructural protein 1 [Influenza A virus (A/Puerto Rico/8-CIP045_RG83841/1934(H1N1))]	↑	64	58%	25823/5.60	cytoplasm	VADQELGDAPFLDR
**J9**	gi|299781726	Influenza A virus	nonstructural protein 1 [Influenza A virus (A/X-53(Puerto Rico/8/1934-New Jersey/11/1976)(H1N1))]	↑	87	62%	25508/6.70	cytoplasm	VADQELGDAPFLDR
**J10**	gi|312436295	Influenza A virus	nonstructural protein 1 [Influenza A virus (A/Puerto Rico/8-CIP045_RG83841/1934(H1N1))]	↑	82	61%	25823/5.60	cytoplasm	VADQELGDAPFLDR
**J12**	gi|312436295	Influenza A virus	nonstructural protein 1 [Influenza A virus (A/Puerto Rico/8-CIP045_RG83841/1934(H1N1))]	↑	56	54%	25823/5.60	cytoplasm	YLTDMTLEEMSR
**N14**	gi|299781726	Influenza A virus (A/X-53(Puerto Rico/8/1934-New Jersey/11/1976)(H1N1))	nonstructural protein 1 [Influenza A virus (A/X-53(Puerto Rico/8/1934-New Jersey/11/1976)(H1N1))]	↑	87	62%	25508/6.70	cytoplasm	VADQELGDAPFLDR
**Other proteins**									
**I18**	gi|304373003	Canis lupus familiaris	uncharacterized protein	↓	69	44%	39642/5.54	extracelluar region	LKENTQHLNVEYER

a The arrow“↑” represents the identified proteins were upregulated and the arrow “↓”represents the identified proteins were downregulated.

In recombinant virus-infected cells, 46 proteins were found to differ from wild-type virus-infected cells, including 9 significantly up-regulated proteins and 36 significantly down-regulated proteins. The identified proteins included 4 cytoskeleton proteins, 22 macromolecular biosynthesis proteins, 5 ubiquitin-proteasome pathway (UPP) proteins, 3 signal transduction proteins, 2 stress response proteins, 5 metabolic process proteins, and 5 other proteins (Table 2).

**Table 2:** List of differentially expressed protein spots in MDCK cells infected with recombinant viruses and PR8-wt virus identified by MALDI-TOF/TOF.

Spot no.	Accession no.	Taxonomy	Description	Variation^a^	Best ion score	Coverage	Theoretical Mr/pI	Localization	Peptides identified
**Cytoskeleton proteins**									
**E01**	gi|73948978	Canis lupus familiaris	vimentin isoform 12	↓	11	45%	53622/5.06	cytoplasm	SLYASSPGGAYATR
**E20**	gi|296202808	Canis lupus familiaris	uncharacterized protein	↓	39	29%	33853/8.31	cytoplasm	SQYEQLAEQNRK
**F07**	gi|359279916	Canis lupus familiaris	keratin 19	↑	53	56%	43858/5.16	cytoplasm	DAEAWFTSR
**F10**	gi|114667511	Canis lupus familiaris	keratin, type I cytoskeletal 10	↑	58	29%	58416/5.09	cytoplasm	HGNSHQGEPR
**Macromolecular biosynthesis**									
**C06**	gi|296234309	Canis lupus familiaris	ruvB-like 2	↓	51	46%	52560/5.42	nucleus	VYSLFLDESR
**C17**	gi|168277712	Canis lupus familiaris	uncharacterized protein	↓	61	39%	48760/5.38	nucleus	NLPLPPPPPPR
**C27**	gi|57108097	Canis lupus familiaris	ATP synthase subunit d, mitochondrial-like	↓	88	81%	18695/5.64	mitochondrion	TIDWVAFGEIIPR
**C31**	gi|5441529	Canis lupus familiaris	splicing factor	↓	34	61%	13350/10.12	nucleus	NPPGFAFVEFEDPR
**C32**	gi|119574194	Homo sapiens	heterogeneous nuclear ribonucleoprotein H1 (H), isoform CRA_b	↓	98	27%	44025/6.47	nucleus	HTGPNSPDTANDGFVR
**C36**	gi|221046076	Homo sapiens	eukaryotic translation initiation factor 3, subunit 12	↓	58	43%	24753/4.76	cytoplasm	YNPENLATLER
**C38**	gi|50815	Homo sapiens	eukaryotic initiation factor 4A-I isoform 1	↓	37	45%	42386/6.56	cytoplasm	GIDVQQVSLVINYDLPTNR
**C41**	gi|221102871	Canis lupus familiaris	Histone H4	↓	47	44%	12972/10.84	nucleus	DNIQGITKPAIR
**D01**	gi|111305821	Homo sapiens	valosin-containing protein	↑	29	38%	91640/5.26	cytoplasm	WALSQSNPSALR
**E02**	gi|74001080	Canis lupus familiaris	T-complex protein 1 subunit theta isoform 1	↓	32	17%	60250/5.47	cytoplasm	FAEAFEAIPR
**E07**	gi|345783337	Canis lupus familiaris	ribonuclease inhibitor	↓	65	48%	47077/4.73	cytoplasm	LENCGLTPASCEDLR
**E13**	gi|50978728	Canis lupus familiaris	serine/threonine-protein phosphatase PP1-alpha catalytic subunit	↓	32	50%	38131/5.94	cytoplasm	ICGDIHGQYYDLLR
**E18**	gi|313678231	Canis lupus familiaris	uncharacterized protein	↓	54	53%	36180/5.44	cytoplasm	SHGVYSCPMVVR
**E23**	gi|73946761	Canis lupus familiaris	charged multivesicular body protein 2a isoform 2	↓	42	27%	25146/5.88	cytoplasm	AEAAASALVDADADLEER
**E24**	gi|4826659	Homo sapiens	F-actin-capping protein subunit beta isoform 1	↓	51	50%	30952/5.69	cytoplasm	DYLLCDYNR
**E37**	gi|55960374	Homo sapiens	transgelin 2	↓	66	54%	21244/7.63	cytoplasm	QMEQISQFLQAAER
**E39**	gi|351699546	Canis lupus familiaris	uncharacterized protein	↓	72	22%	17633/5.38	mitochondrion	AQSELSGAADEASR
**E40**	gi|296197159	Canis lupus familiaris	histone H2B	↓	39	58%	13912/10.31	nucleus	EIQTAVR
**F03**	gi|330723765	Canis lupus familiaris	Keratin 12	↑	119	50%	43875/5.78	cytoplasm	NLQSAMAGDNAGVLLR
**F05**	gi|146674809	Canis lupus familiaris	40S ribosomal protein SA	↑	99	37%	25646/5.37	cytoplasm	AIVAIENPADVSVISSR
**F14**	gi|345780308	Canis lupus familiaris	γ-glutamylcyclotransferase	↑	56	46%	21374/5.36	cytoplasm	NPSAAFCCVAR
**F15**	gi|57088081	Canis lupus familiaris	transcription elongation factor B polypeptide 2 isoform 1	↑	41	59%	12613/5.01	nucleus	ADEAFEALR
**Ubiquitin-proteasome pathway**									
**C08**	gi|4506209	Homo sapiens	26S protease regulatory subunit 7 isoform 1	↓	49	42%	49002/5.71	cytoplasm	SVCTEAGMFAIR
**C26**	gi|351699703	Homo sapiens	Ubiquitin-conjugating enzyme E2 N	↓	48	21%	16048/5.19	nucleus	DKWSPALQIR
**E06**	gi|296218057	Canis lupus familiaris	uncharacterized protein	↓	58	54%	49899/5.24	cytoplasm	DSYLILETLPTEYDSR
**E33**	gi|345782656	Canis lupus familiaris	proteasome subunit beta type-4	↓	70	46%	28796/5.48	cytoplasm	QVLGQMVIDEELLGDGHSYSPR
**F16**	gi|351699703	Canis lupus familiaris	uncharacterized protein	↑	48	19%	16048/5.19	nucleus	DKWSPALQIR
**Signal transduction**									
**C19**	gi|55742853	Canis lupus familiaris	annexin A4	↓	107	32%	36075/5.72	cytoplasm	GGTVKPASGFSATEDAQTLR
**C35**	gi|345786899	Canis lupus familiaris	abhydrolase domain-containing protein 14B	↓	47	36%	22412/5.62	cytoplasm	AVAIDLPGLGR
**F09**	gi|73946797	Canis lupus familiaris	annexin A1	↑	74	20%	38887/5.84	cytoplasm	TPAQFDADELR
**Stress response**									
**A09**	gi|359322074	Canis lupus familiaris	peroxiredoxin-2-like isoform 1	↓	92	37%	22112/5.23	cytoplasm	SLSEDYGVLKEDEGIAYR
**C01**	gi|296474454	Canis lupus familiaris	uncharacterized protein	↓	74	33%	78895/5.18	mitochondrion	HFSVEGQLEFR
**Metabolism associated proteins**									
**B04**	gi|188590	Canis lupus familiaris	Histone H2B	↑	48	51%	15214/4.56	cytoplasm	DQGTYEDYVEGLR
**C12**	gi|345778725	Canis lupus familiaris	lactoylglutathione lyase	↓	39	50%	20982/5.26	cytoplasm	DFLLQQTMLR
**C14**	gi|57088769	Canis lupus familiaris	N(G),N(G)-dimethylarginine dimethylaminohydrolase 1	↓	28	27%	31629/5.94	cytoplasm	AQGDEVDFAR
**E27**	gi|356460954	Canis lupus familiaris	chloride intracellular channel protein 1	↓	39	50%	25944/5.39	plasma membrane	GFSIPEVFR
**E34**	gi|345778725	Canis lupus familiaris	lactoylglutathione lyase	↓	57	50%	20982/5.26	cytoplasm	DFLLQQTMLR
**Other and unknown proteins**									
**C16**	gi|304373003	Canis lupus familiaris	uncharacterized protein	↓	69	36%	39642/5.54	extracelluar region	LKENTQHLNVEYER
**C21**	gi|345779526	Canis lupus familiaris	uncharacterized protein	↓	22	36%	28983/5.08	cytoplasm	AAAPQTQHVSPMR
**C28**	gi|73955331	Canis lupus familiaris	subcomponent-binding protein, mitochondrial	↓	45	32%	30417/4.77	mitochondrion	EVSFQAVGESEWK
**D05**	gi|355557693	Canis lupus familiaris	Stathmin	↑	68	36%	17229/5.76	cytoplasm	ASGQAFELILSPR

a The arrow“↑” represents the identified proteins were upregulated and the arrow “↓”represents the identified proteins were downregulated.

Several differentially expressed proteins were involved in macromolecular biosynthesis, including histone H4 (spot C41), which was down-regulated in all three groups of recombinant virus-infected cells. There were two down-regulated proteins in cells infected with rPR8-H5N1NA and rPR8-H9N2NA compared to cells infected with PR8-wt: ruvB-like 2 (RUVBL2, spot C06, spot E05) and annexin A4 (spot C19, spot E22). Twenty-four differentially expressed proteins were identified in rPR8-H5N1NA-infected MDCK cells compared with PR8-wt.

The identified proteins included several other species, such as *Homo sapiens*, because of the missing dog proteins in the database and the uncharacterized proteins of *Canis lupus familiaris*, which showed high homology to proteins of other species.

### The possible fusion-related proteins in host cells

Cells infected with rPR8-H5N1NA and rPR8-H9N2NA recombinant viruses can induce more cell-cell fusion than rPR8-H1N1NA and PR8-wt viruses [5]. This result suggested that the differentially expressed proteins in cells infected with rPR8-H5N1NA or rPR8-H9N2NA virus may be involved in cell-cell fusion. Therefore, we investigated the functions of differentially expressed proteins.

Compared to cells infected with the wild-type virus, RUVBL2 (RuvB-like 2, spot C06) and charged multivesicular body protein 2a isoform 2 (CHMP2A, spot E23) proteins were down-regulated in cells infected with rPR8-H5N1NA or rPR8-H9N2NA. RUVBL2 (spot C06) is an AAA ATP enzyme family member that functions in a wide range of cellular processes, including chromatin remodeling and transcriptional regulation [8]. RUVBL2 is a critical mediator of MLL-AF9-induced oncogenesis, which has been shown to induce leukemia in mice and humans. The down-regulation of RUVBL2 may be a host defense response against cell fusion. CHMP2A (spot E23) is an endosomal sorting complex (ESCRT-0-III) for transport that allows budding from the cytoplasm or membrane fission. It is involved in multivesicular endosome synthesis, cytokinesis, and the budding of some membrane proteins. Membrane fission is catalyzed by ESCRT-III complexes composed of CHMP polymers. Down-regulation of spot E23 may be a host response to prevent cytoplasmic fission or cytoplasmic spillover.

Compared to PR8-wt infected cells, F-actin-capping protein subunit beta isoform 1 (spot E24) was downregulated in rPR8-H5N1NA-infected cells. The capping protein is a heterodimer that can prevent actin filament assembly and disassembly at the fastest growing actin end and modulates actin filament dynamics while stabilizing the length of actin filaments in muscle and non-muscle tissues. The 40 S ribosomal protein SA (RPSA, spot F05) was upregulated after H5N1 infection compared to PR8-wt virus infection. RPSA is alternatively called the laminin receptor-1 (LamR). The interaction between LamR and laminin helps viruses attach to tumor cell plasma membranes, including Sindbis virus [9]-[12]. This type of interaction could mediate changes in the cell microenvironment to impact cell adhesion, tumor growth, and metastasis, and RPSA is required for maintaining cell viability [13]. Our observation that RPSA levels increase in rH5N1NA-infected MDCK cells suggests that RPSA may facilitate cell fusion.

CHMP2A isoform 2 (spot E23) and F-actin-capping protein subunit beta isoform 1 (spot E24) are related to cell membrane fission and fiber assembly. RUVBL2 (spot C06) and 40 S ribosomal protein SA (spot F05) are related to tumor formation and cell viability. Previous studies showed that cell-cell fusion in MDCK cells infected with rPR8-H5N1NA occurs more quickly and more frequently than in wild-type virus-infected cells [5].

### Transcriptional profiles of differentially expressed proteins following infection

**Figure 3:**
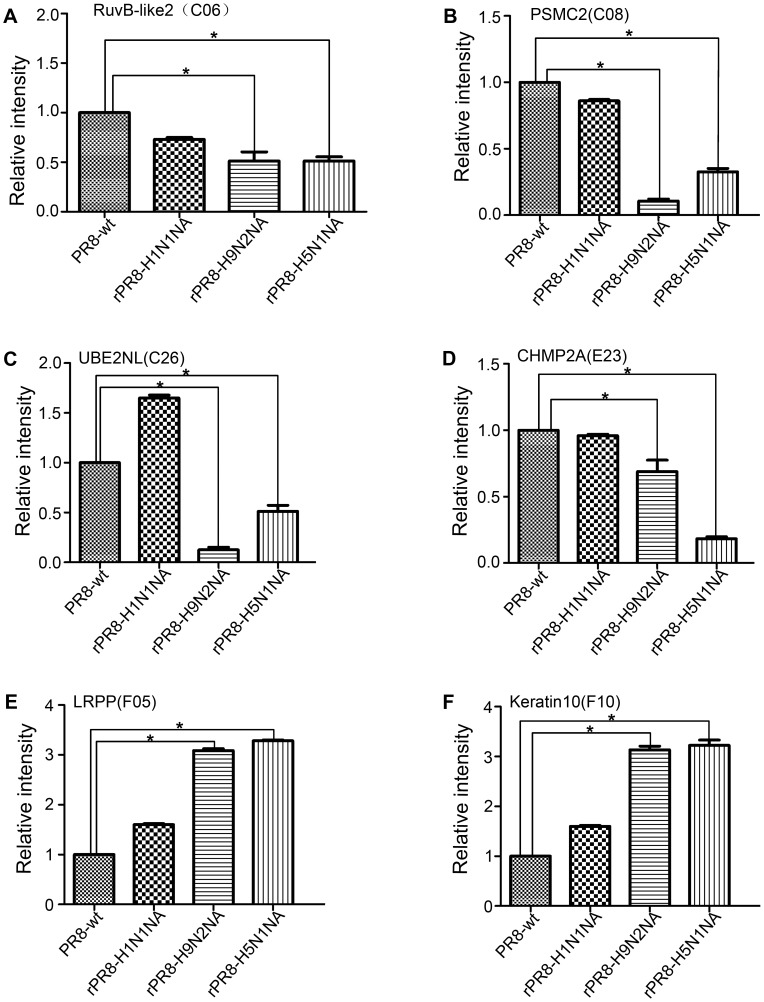
Transcriptional profiles of differentially expressed proteins in influenza virus-infected MDCKs. Total cellular RNA from MDCKs with or without influenza virus infection was subjected to real-time RT-PCR. Samples were normalized to mock-infected MDCKs using β-actin as the reference gene.

Six genes were selected from the differentially expressed protein list to analyze transcriptional alterations, with the β-actin gene serving as a control. The trends in alterations of mRNA abundance levels of these six genes in recombinant virus-infected and wild-type virus-infected cells were similar to changes observed in the patterns of corresponding proteins on 2-DE gels. The mRNA abundance of RUVBL2 (spot C06), PSMC2 (spot C08), CHMP2A isoform 2 (spot E23), and UBE2NL (spot C26) were down-regulated compared to levels in wild-type infected cells, whereas the 40 S ribosomal protein SA (spot F05) and keratin 10 (spot F10) genes were up-regulated (Fig. 3). These data support the results of the proteomics analysis.

### Western blot and IIFA validation

Ubiquitylation modifications catalyzed by specific ubiquitin enzymes could induce the highly selective degradation of specific signal proteins that play important roles in maintaining normal cellular functions. The expression of 26 S protease regulatory subunit 7 isoform 1 (spot C08) and ubiquitin-conjugating enzyme E2N (spot C26) in cells infected with PR8-wt were remarkably up-regulated compared to rPR8-H9N2NA-infected cells.

**Figure 4:**
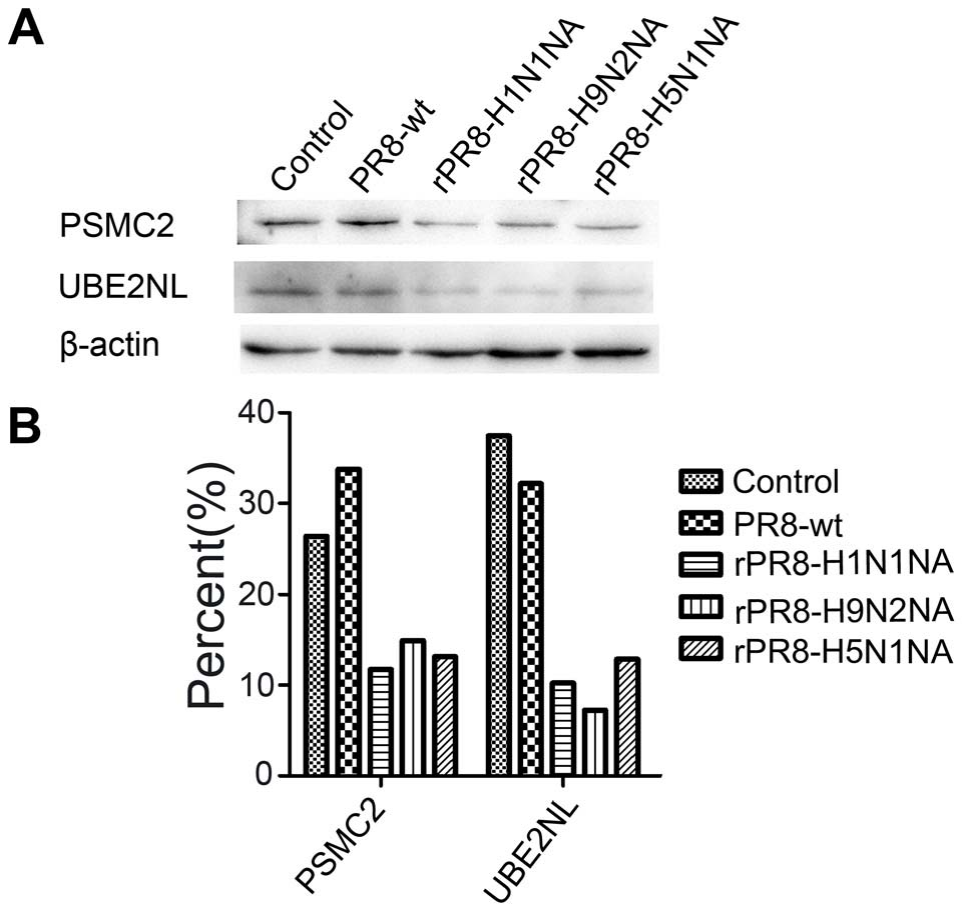
Western blots of representative proteins in influenza virus-infected MDCKs. The samples were prepared from MDCK cells that were virus-infected or mock-infected cells at 6 h p.i.. The β-actin protein was used as a control. (A) Western blot confirmation of differentially expressed proteins for PSMC2 (C08) and UBE2NL (C26). (B) ImageJ software analysis of the ratios of proteins changes according to Fig. 4A.

To further confirm protein expression alterations during influenza virus infection, these two proteins were assessed with western blot analyses. Equal amounts of lysates from influenza virus-infected and control cells were examined with specific antibodies to β-actin, UBE2NL (spot C26), and PSMC2 (spot C8). Western blot analyses revealed that UBE2NL and PSMC2 levels in cells infected with recombinant viruses were down-regulated compared with PR8-wt-infected cells (Fig. 4), which is consistent with the proteomics results.

**Figure 5:**
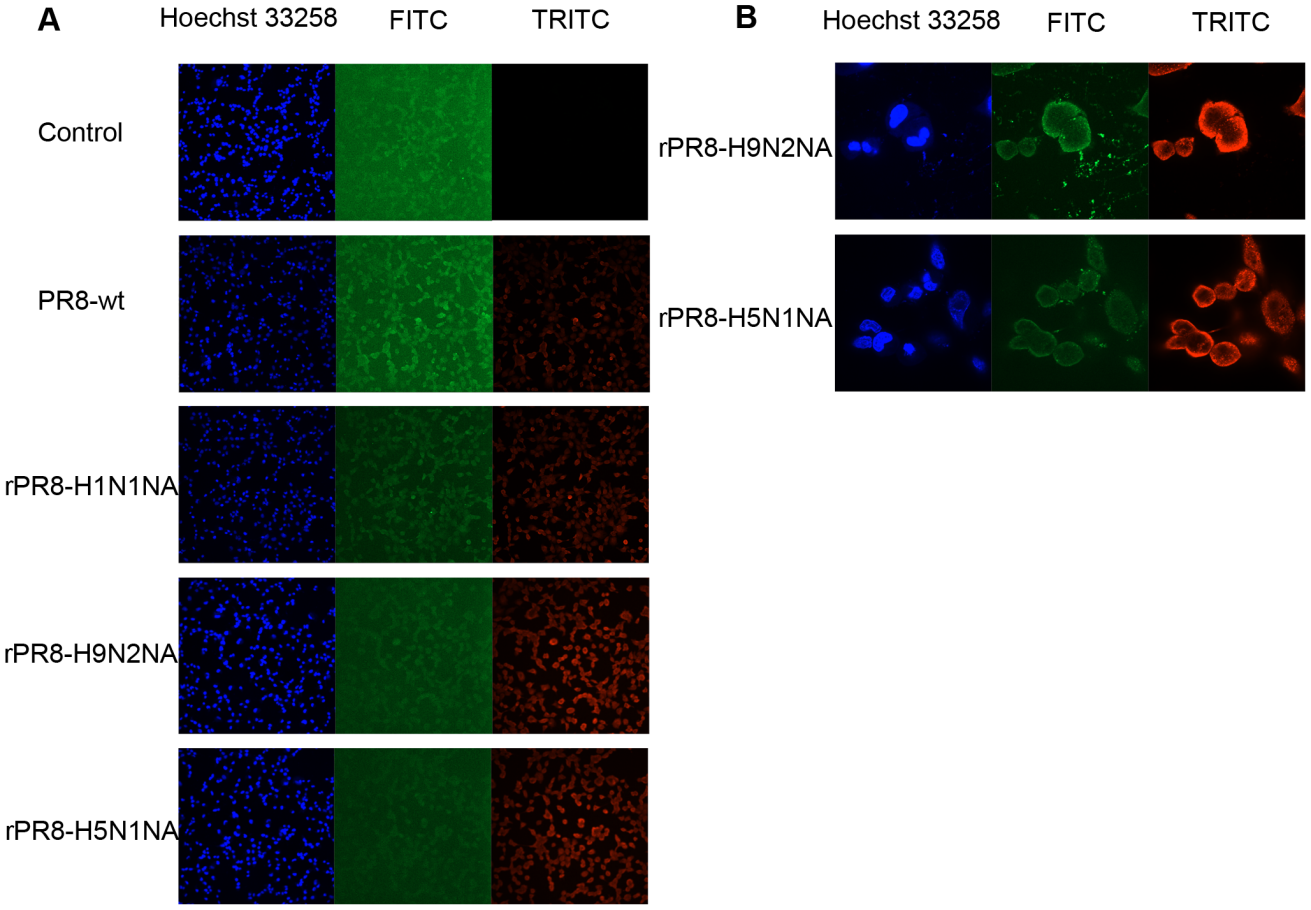
UBE2NL protein in virus-infected or mock-infected MDCK cells at 6 h p.i.. MDCK cells were infected with the viruses at MOI of 0.1 in the presence of 1 µg/ml TPCK-trypsin. After adsorption for 1 h at 37°C, the inocula were removed and the cultures were incubated for 6 h at 37°C in the maintenance media. Then, the cells were processed for indirect immunofluorescence assay, and the infected cells were detected with polyclonal antisera to UBE2NL protein and NP protein. (A) The fluorescence images (10×) of the infected and mock-infected cells at 6 h p.i. The FITC-fluorescence signal was expressed as UBE2NL protein and TRITC-fluorescence signal was expressed as the infected cells. (B) The fluorescence images (60×) of the cells infected by rPR8-H9N2NA or rPR8-H5N1NA viruses.

To verify whether the fusion phenotype is dependent on the UBE2NL protein, an indirect immunofluorescence assay was performed using the polyclonal antibody to UBE2NL or NP protein of influenza virus. As shown in Fig. 5A, the UBE2NL protein distribution were the whole-cell pattern and the cell infected by PR8-wt or mock-infected showed higher FITC positive rates than those infected by rPR8-H9N2NA and rPR8-H5N1NA. But the virus infection process of rPR8-H9N2NA or rPR8-H5N1NA viruses were more extensively than that of PR8-wt. The fused cells with the nucleuses gathered together were shown in Fig 5B. The data demonstrated that the fusion phenotype was related to the UBE2NL protein.

One of the common obstacles a virus faces is the overwhelming stoichiometric imbalance of host target proteins to viral proteins upon initial infection. To overcome this, many viruses have evolved a mechanism whereby their cellular target-proteins are directed to the 26S proteasome and subjected to proteolytic degradation [14]. E2 ubiquitin-conjugation has been proven to be a key mediator of the ubiquitin-proteasome [15]–[18]. These data demonstrate that wild-type viruses have evolutionary advantages. It is clear that the wild-type virus causes reduced alteration of gene expression, which is potentially advantageous for the wild-type virus in terms of escaping detection. In contrast, the recombinant viruses caused extensive intracellular changes, which we hypothesize to be representative of an evolutionary disadvantage due to an imbalanced replication program. These changes can be explained by one reason: recombinant viruses with NA replacements are not wild-type or pandemic viruses in nature, and the eight genome segments of recombinant virus may not be complete matches, so intracellular protein expression undergoes extensive changes although it should be noted that in our in vitro system the virus titers of recombinant and wild-type virus are equivalent. Therefore, our data provide evidence for virus evolution in nature.
